# Iron-Handling, Lipid-Oxygenation, and Hypoxia-Response Gene Expression in the Renal Cortex of Cats with Chronic Kidney Disease: An Analysis-Plan-Guided Secondary Analysis

**DOI:** 10.3390/vetsci13060604

**Published:** 2026-06-22

**Authors:** Cleverson de Souza

**Affiliations:** Department of Comparative, Diagnostic, and Population Medicine, College of Veterinary Medicine, University of Florida, Gainesville, FL 32610, USA; cdesouza@ufl.edu

**Keywords:** feline chronic kidney disease, iron metabolism, lipid oxygenation, hypoxia signaling, renal cortex, RNA sequencing

## Abstract

Chronic kidney disease (CKD) is common in older cats, but cortical transcript-level relationships among hypoxia response, iron handling, and lipid oxygenation are poorly defined. This analysis-plan-guided, hypothesis-generating secondary analysis used public feline renal RNA-seq data (GSE303653). The internal plan fixed the gene panel, composite construction, primary inferential test, and quality-control thresholds before the review of the present expression results, but was not publicly registered. After technical quality control, 21 renal cortex samples from control, CKD 1/2, and CKD 3/4 cats were analyzed. A 23-gene panel and whole-transcriptome differential expression were evaluated using likelihood ratio testing as the primary panel-level screen, with pairwise DESeq2 contrasts, Spearman summaries, enrichment, medulla, composite, and marker-set analyses as secondary or exploratory context. VEGFA, FTL, and NCOA4 decreased with ordinal disease group, whereas ALOX5 and HIF1A increased; eight panel genes were stage-associated by likelihood ratio testing. The equal-weight composite was nonmonotonic. Advanced CKD enrichment was dominated by immune and inflammatory terms, while GPX4 and ferroptosis-pathway enrichment were not stage-significant. The findings support heterogeneous transcript-level remodeling, including ALOX5-associated inflammatory/lipid-oxygenation signal and HIF1A–VEGFA divergence, rather than evidence of ferroptotic cell death, pathway activation, or cell-specific mechanism.

## Simple Summary

Chronic kidney disease is common in older cats and often worsens over time, but the tissue changes behind the disease are not fully understood. This study reanalyzed public gene expression data from the kidney cortex, the outer part of the kidney, from cats without kidney disease and cats with mild or more advanced chronic kidney disease. We focused on genes related to low oxygen, iron handling, and fat-related injury signals because these processes may help explain how kidney tissue changes during disease. Several genes changed as disease severity increased, but they did not move together as one simple pathway. Instead, the results suggested mixed inflammation, low oxygen response, iron handling, and tissue remodeling changes. The findings do not prove a specific form of cell death or identify the exact cell types involved. They do provide candidate patterns that can be tested in future studies using tissue imaging, protein measurements, and cell level methods. This work helps narrow down which kidney pathways deserve deeper study in cats with chronic kidney disease.

## 1. Introduction

Chronic kidney disease (CKD) is common in older cats [1]. Longitudinal work in geriatric cats has identified risk factors for CKD development [2]. Clinical staging in cats is commonly based on International Renal Interest Society (IRIS) criteria [3]. In feline renal tissue, naturally occurring CKD has been associated with increased HIF1A transcription and decreased VEGFA transcription [4]. Integrated multi-omics has since shown broader cortical and medullary remodeling with hypoxia-related, metabolic, and inflammatory signals in feline CKD [5]. Hypoxia-related mechanisms and their relevance to cats have been reviewed [6]. What remains unresolved is narrower than a general CKD transcriptomics gap: in the publicly available feline kidney RNA-seq dataset, it is unclear whether the renal cortex shows consistent stage-associated relationships among hypoxia-response, iron-handling, and lipid-oxygenation genes, or whether bulk-tissue changes are better explained as heterogeneous remodeling across inflammatory, tubular, endothelial, and fibrotic compartments.

These gene families are related, but they are not equivalent biological readouts. Ferroptosis is a regulated cell-death process most convincingly supported by convergent evidence for iron-dependent lipid peroxidation, loss of glutathione peroxidase 4 activity, and compatible injury phenotypes [7,8,9,10]. By contrast, HIF1A, VEGFA, HMOX1, ALOX5, ferritin genes, and iron-transport genes are also broadly involved in oxidative stress, inflammation, tissue remodeling, hypoxia response, and renal injury. Renal hypoxia signaling is not a uniform transcript-level output: HIF-1alpha and HIF-2alpha occupy different renal cell compartments [11], progressive human kidney disease can show hypoxia-response signaling despite reduced VEGF-A expression [12], and renal oxygen sensing is governed largely by HIF hydroxylation and protein stabilization rather than by HIF transcript abundance alone [13]. Ferritinophagy can mobilize ferritin-bound iron through NCOA4 [14], 5-lipoxygenase signaling can contribute to renal fibrosis and CKD progression [15], and heme oxygenase-1 induction can be cytoprotective in renal injury models [16]. These links justify examining the genes together, but they do not justify treating bulk renal cortex transcript abundance as direct evidence of ferroptotic cell death, pathway activation, or cell-specific mechanisms.

Our objective was to determine whether the expression of genes related to iron handling, lipid oxygenation, and hypoxia response differs across ordinal disease groups in the feline renal cortex. We used a 23-gene analysis-plan-defined panel together with a whole-transcriptome context to evaluate whether these genes changed coherently or showed divergent transcript-level patterns. We hypothesized that at least some panel genes would differ by ordinal disease group. We treated the equal-weight composite as an analysis-plan-defined descriptive summary, not as a primary biologic endpoint or as a test of ferroptotic cell death.

## 2. Materials and Methods

This was an analysis-plan-guided cross-sectional secondary analysis of publicly available feline renal RNA-sequencing data from GSE303653 [5]. Reporting followed STROBE guidance [17]. The quality-control exclusion thresholds, 23-gene cortical panel, equal-weight composite definition, primary inferential test, and planned sensitivity analyses were documented in an internal statistical analysis plan before the review of the present expression results (Supplementary Document S1). The plan was not deposited in a public registry; therefore, the term analysis-plan-guided is used to indicate protection against ad hoc analytic drift within this project, not externally verifiable preregistration. The primary analysis used the renal cortex because CKD 3/4 medulla samples were limited; medulla results were summarized descriptively as exploratory only.

The analytical hierarchy was defined as follows. The primary inferential analysis was the DESeq2 likelihood ratio test (LRT) for disease-group association within the 23-gene cortical panel. Secondary analyses were pairwise DESeq2 Wald contrasts and Spearman ordinal trend summaries for panel genes. Exploratory analyses included whole-transcriptome enrichment, medulla summaries, equal-weight composite sensitivity analyses, and bulk marker-set summaries used to contextualize possible cell-composition effects. This hierarchy was added to make clear that the study was not designed to infer ferroptotic cell death, pathway activation, disease progression trajectories, or cell-specific mechanisms from bulk RNA-seq.

Two samples were excluded before hypothesis testing because they met technical quality-control failure criteria fixed in the analysis plan (mapping rate < 35% OR duplication > 70% OR robust within-tissue principal-component-analysis outlier with Mahalanobis distance *p* < 0.01). These cutoffs were chosen as conservative bounds that nf-core/rnaseq runs on moderate-depth tissue RNA-seq are expected to meet by a wide margin; samples falling outside them typically reflect technical failures such as degradation, library bias, or mislabeling rather than interpretable biological variation. PCA was used only as a technical outlier screen within tissue, not as evidence for downstream biological interpretation. The exclusions comprised SRR34712210, a CKD 1/2 cortex sample, and SRR34712180, a CKD 3/4 medulla sample. After applying these criteria, the primary analytic set contained 21 cortex samples from 21 cats: 6 controls, 8 CKD 1/2, and 7 CKD 3/4. Stage assignments were taken from the source study, which classified cats by IRIS stage [3,5].

The cortical panel included 23 genes chosen to represent intersecting, but non-equivalent, biology: iron storage and transport, ferritin handling/ferritinophagy, lipid oxygenation and lipid-peroxidation defense, heme/redox handling, and hypoxia-response/oxygen-sensing transcripts. The panel was intended to test whether these transcript groups behaved coherently in bulk cortex, not to define a validated ferroptosis signature. The original statistical analysis plan defined an equal-weight composite of the two gene arms; this earlier terminology is retained only when referring to the analysis plan or figures that reproduce those analyses. The composite itself is interpreted descriptively because the iron-handling/lipid-oxygenation and hypoxia-response arms moved in different directions across ordinal disease groups, making gene-level results more informative than the averaged summary.

Read processing and transcript quantification followed the nf-core/rnaseq workflow (v3.16.1) [18] with Salmon (v1.10.1) pseudo-alignment against the Felis catus 9.0 reference (Ensembl release 113), as detailed in Appendix A [19]. Transcript-level abundances were imported into R with tximeta v1.20.1, length-scaled (countsFromAbundance = lengthScaledTPM), and aggregated to gene level using the GTF gene_id attribute; DESeq2 v1.44.0 independent filtering was applied to remove low-count genes before pairwise Wald tests and the omnibus LRT. We used DESeq2 because it is a widely used negative-binomial framework for count-based RNA-seq differential expression and because the goal was a transparent secondary analysis rather than comparison of statistical engines [20]. Alternative frameworks such as edgeR or limma-voom could affect borderline genes and are therefore treated as a limitation rather than as a separate set of confirmatory analyses.

Gene-level stage associations were assessed with Spearman correlations and bootstrap confidence intervals. Spearman correlations with a 3-level ordinal predictor produce substantial tied ranks and should be interpreted as descriptive group-separation summaries rather than continuous progression models. We refer to this predictor as ordinal disease group (control, CKD 1/2, CKD 3/4) rather than as CKD stage, because controls are not stage 0 and IRIS stages 1/2 and 3/4 are collapsed in the source classification. Whole-transcriptome differential expression used DESeq2 with 3 pairwise Wald contrasts (default unshrunken maximum-likelihood log2 fold change estimates) and an LRT for stage-dependent expression [20]. Pairwise differential expression required Benjamini–Hochberg-adjusted *p* < 0.05 and |log2 fold change| > 1 within each contrast. The fold-change filter was used as an effect-size threshold for reporting, not as evidence that genes below that threshold lacked biological relevance.

These choices differ from the source-study report by Li et al. [5] in three respects: Li used edgeR’s glmQLFTest rather than DESeq2, applied a Benjamini–Hochberg threshold of 0.10 rather than 0.05, and did not document use of a fold-change filter. Under our DESeq2 plus Benjamini–Hochberg *p* < 0.05 criterion without the |log2 fold change| > 1 reporting threshold, the cortex DEG count for CKD 3/4 versus controls was 4560, differing by 56 genes (about 1.2%) from Li’s reported 4616 and consistent with the two-sample technical exclusion described above. The CKD 1/2 versus controls discrepancy (539 here vs. 6 in Li) is larger and reflects, at minimum, the different statistical engine and threshold; the smaller early-stage contrast is therefore interpreted more cautiously.

Functional enrichment used g:Profiler with the g:SCS multiple-testing correction [21]. Because cat-specific GO and pathway databases are sparse, gene sets were mapped to their human orthologs (HGNC symbols) and queried against the Homo sapiens reference, with the 16,959 cortex-tested genes used as the custom background to avoid genome-wide-default bias. Ortholog/query retention was high for the DEG sets used for enrichment: 535 of 539 CKD 1/2 versus control DEGs (99.3%), 2678 of 2686 CKD 3/4 versus control DEGs (99.7%), and 249 of 250 CKD 3/4 versus CKD 1/2 DEGs (99.6%) were retained for g:Profiler queries (Table S2). To contextualize possible cell-composition shifts, mean z-scored log2(counts per million + 1) expression of conserved cell-type marker sets (macrophage/monocyte, proximal tubule, endothelial, fibroblast/myofibroblast, and cytokine/NF-kappaB) was computed per cortex sample and summarized by ordinal disease group (Table S7). These marker-set analyses were exploratory context only and were not used as formal cell-type deconvolution. Composite summaries are reported as medians, interquartile ranges, means, standard deviations, and Hedges’ g with bootstrap confidence intervals as descriptive secondary analyses. A formal LRT-based power calculation was not performed because LRT power for transcriptome-wide RNA-seq is dispersion-dependent and not summarized by a single effect-size threshold; the closest available power statement applies to the panel-level Spearman analyses (see Discussion). Additional processing details, sensitivity analyses, exploratory medulla summaries, and the cell-composition marker analysis are provided in Appendix A and Supplementary Tables S1–S7.

## 3. Results

### 3.1. Sample Characteristics and Quality Control

Source-study cohort 2 comprised renal tissues from 23 cats [5]. Review of the public GSE303653 deposit identified 40 renal tissue samples, of which 38 samples from 22 cats passed technical quality-control criteria. Two samples were excluded before hypothesis testing because they met the analysis-plan-defined failure thresholds described in Materials and Methods: one medulla sample with mapping rate 21.6% and duplication 80.6%, and one cortex sample with mapping rate 32.2%, duplication 73.5%, and principal-component-analysis outlier status. The retained set comprised 21 cortex samples for the primary analysis and 17 medulla samples for exploratory summaries; the GSE303653 deposit listed 22 cortex-labeled and 18 medulla-labeled RNA-seq records (40 total), and the source publication’s report of 21 cortical samples corresponds to the analytic set after the technical exclusion described above. Source-cohort demographic information reported by Li et al. is shown in Table 1 [5].

### 3.2. Gene-Level Stage Associations Within the Cortical Panel

**Figure 1:**
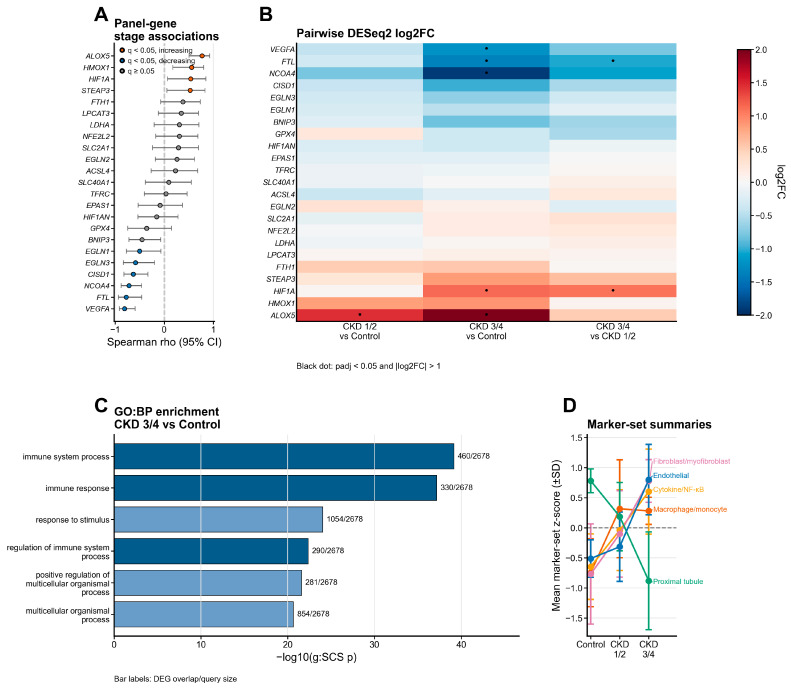
Summary of gene-level and whole-transcriptome context by ordinal disease group in feline renal cortex. (**A**) Forest plot of Spearman rho values with 95% confidence intervals for the 23-gene cortical panel; orange and blue indicate genes increasing or decreasing with ordinal disease group at Spearman q < 0.05, respectively, and gray indicates q ≥ 0.05. (**B**) Heatmap of pairwise DESeq2 log2 fold changes for panel genes; black dots indicate adjusted *p* < 0.05 and |log2 fold change| > 1. (**C**) Top enriched GO:BP terms among CKD 3/4 versus control differentially expressed genes; bars show −log10(g:SCS *p*), and labels show DEG overlap/query size. (**D**) Exploratory marker-set summaries from conserved marker groups; points show mean z-scored log2(CPM+1) marker-set score by ordinal disease group, and error bars show standard deviation. Panels are generated from Supplementary Tables S1, S2a, S5, and S7.

Gene-level analyses were more informative than the original equal-weight composite. In the cortical panel, the strongest decreasing stage associations were VEGFA (correlation coefficient, −0.81; 95% confidence interval [CI], −0.92 to −0.59), FTL (−0.78; 95% CI, −0.93 to −0.46), and NCOA4 (−0.72; 95% CI, −0.88 to −0.46). The strongest increasing associations were ALOX5 (0.77; 95% CI, 0.50 to 0.93), HMOX1 (0.56; 95% CI, 0.17 to 0.80), HIF1A (0.54; 95% CI, 0.07 to 0.85), and STEAP3 (0.53; 95% CI, 0.05 to 0.83; Table 2; Figure 1A; full panel in Table S1). After Benjamini–Hochberg correction across the 23 panel-level Spearman tests, 10 of 11 nominally significant correlations were retained at q < 0.05; BNIP3 (raw *p* = 0.040, q ≈ 0.082) was the gene that fell below the BH threshold (Table S1). Eight of 23 panel genes were also stage-dependent by likelihood ratio testing using the same Benjamini–Hochberg adjustment within the LRT family: VEGFA, HIF1A, FTL, NCOA4, STEAP3, EGLN1, ALOX5, and EGLN3. CISD1 and HMOX1 reached significance only on the secondary Spearman screen and not on the primary likelihood ratio test, and were therefore treated as weaker, exploratory candidates. By contrast, GPX4 and EPAS1 showed little stage association (correlation coefficients, −0.36 and −0.09, respectively; Table S1). GPX4 did not meet panel-level Spearman, likelihood ratio test, or pairwise differential-expression thresholds in any contrast. ALOX5 was the only panel gene meeting the differential-expression threshold in CKD 1/2 versus controls, whereas ALOX5, HIF1A, FTL, NCOA4, and VEGFA met that threshold in CKD 3/4 versus controls. HIF1A and FTL differed between CKD 3/4 and CKD 1/2 (Figure 1B).

### 3.3. Whole-Transcriptome Differential Expression and Pathway Context

Whole-transcriptome differential expression increased with disease severity. CKD 1/2 versus control yielded 539 differentially expressed genes (DEGs), CKD 3/4 versus control yielded 2686, and CKD 3/4 versus CKD 1/2 yielded 250 (Table S2). Functional enrichment of CKD 3/4 versus control DEGs was dominated by immune and inflammatory biology, including immune system process and immune response, with reactive oxygen species metabolic process appearing among less dominant significant terms (Figure 1C; Table S5). A ferroptosis-specific pathway was not significantly enriched. Exploratory bulk marker-set summaries were used to contextualize possible cell-composition effects (Figure 1D; Table S7): macrophage/monocyte markers were elevated in both CKD 1/2 and CKD 3/4 relative to controls, fibroblast/myofibroblast and cytokine/NF-κB marker sets increased with ordinal disease group, and proximal-tubule markers decreased sharply in CKD 3/4. These summaries were exploratory and were not used as formal deconvolution.

### 3.4. Analysis-Plan-Defined Composite Summary

The analysis-plan-defined equal-weight cortical composite was a less informative summary than the underlying gene-level results (Table 3; Figure S1). The composite did not change monotonically with stage (median values 0.20 for controls, −0.09 for CKD 1/2, 0.26 for CKD 3/4), per-gene stage associations were heterogeneous within both panel arms (Figure S2), and all three pairwise composite effect sizes had bootstrap 95% confidence intervals crossing zero (Table 4). Sensitivity analyses, including leave-one-out exclusion of a single technically retained sample with an extreme score, did not change this central interpretation; composite sensitivity summaries are reported in Tables S3 and S6.

### 3.5. Exploratory Medulla Summary

Exploratory medulla summaries did not mirror the cortex pattern exactly, although interpretation was limited by the small number of CKD 3/4 medulla samples (n = 4). Median medulla composite values were −0.04 for controls, 0.20 for CKD 1/2, and 0.01 for CKD 3/4 (Table S4).

## 4. Discussion

The central finding of this cortical RNA-sequencing analysis was heterogeneous transcript-level remodeling rather than uniform activation of a single ferroptosis-related program. ALOX5, HIF1A, HMOX1, and STEAP3 increased with ordinal disease group, whereas VEGFA, FTL, NCOA4, EGLN1, and EGLN3 decreased. Because the original equal-weight composite averaged genes moving in opposite directions, it performed poorly as a summary statistic. The most informative result is therefore the split behavior of individual genes within the 23-gene panel, not the composite itself.

This pattern is compatible with, but more restricted than, the existing feline literature. Lourenço and colleagues reported higher HIF1A and lower VEGFA transcript levels in kidneys from cats with naturally occurring CKD than in controls [4]. Li and colleagues then documented broader cortical and medullary remodeling with hypoxia-related, metabolic, and inflammatory signals in feline CKD by integrated multi-omics [5]. The present analysis differs from Li et al. in three main ways: it emphasizes renal cortex, applies an analysis-plan-defined 23-gene panel focused on iron handling, lipid oxygenation, and hypoxia response, and evaluates broad ordinal disease groups rather than CKD versus control alone. Methodological differences also help explain why differential-expression counts differed between studies, especially for the CKD 1/2 comparison. For that reason, the present results should be read as a cautious secondary analysis of the public dataset rather than as a replacement for the source-study transcriptomic interpretation.

The ferroptosis interpretation should be deliberately narrow. Experimental kidney disease models support ferroptosis most convincingly when glutathione peroxidase 4 is suppressed, lipid-oxygenation injury is demonstrated directly, and ferritinophagy-linked iron mobilization is supported [8,9,10,22]. In the present dataset, GPX4 was not stage-associated, ferroptosis-pathway enrichment was not significant, and NCOA4 decreased rather than increased. Reduced NCOA4 and FTL expression may reflect altered ferritin handling, loss or remodeling of ferritin-rich tubular populations, inflammatory replacement, or a combination of these processes; bulk transcript abundance alone cannot distinguish among these explanations. Recent feline biomarker work reporting increased urine GPX4 in cats with CKD reflects ongoing interest in this pathway [23], but the present tissue-level transcriptomic results do not provide evidence sufficient to claim ferroptotic cell death or a classical ferroptosis-driven model.

ALOX5 is better interpreted as an inflammatory/lipid-oxygenation signal than as a specific ferroptosis readout. In renal disease models, 5-lipoxygenase contributes to fibrosis and CKD progression, and macrophages can be an important renal source of 5-lipoxygenase expression [15]. In this dataset, ALOX5 was higher in both CKD 1/2 and CKD 3/4 than in controls, while whole-transcriptome enrichment in CKD 3/4 was dominated by immune and inflammatory terms. However, bulk RNA-seq cannot determine whether higher ALOX5 reflects inflammatory-cell abundance, transcriptional activation within infiltrating or resident cells, or both. HMOX1 should be interpreted with similar caution because heme oxygenase-1 can be cytoprotective and antifibrotic in renal injury models [16]. These findings are most appropriately framed as transcript-level evidence of inflammatory/lipid-oxygenation and redox remodeling, not as proof of a cell-specific mechanism.

The hypoxia-related result is best described as transcript-level divergence between HIF1A and VEGFA. HIF1A transcript abundance increased with ordinal disease group, whereas VEGFA decreased sharply and EPAS1 showed little stage association. Because renal oxygen sensing is governed largely by HIF hydroxylation and protein stabilization, rising HIF1A messenger RNA should not be equated with uniform HIF pathway activation [13]. HIF-1α and HIF-2α also occupy different renal cell compartments [11], and progressive human kidney disease can show hypoxia-response signaling despite reduced VEGF-A expression [12]. Recent feline work reported that plasma HIF-1α protein concentrations increased in early CKD but declined in advanced stages [24], whereas HIF1A transcript abundance increased across ordinal disease groups in the present analysis. This cross-study difference is compatible with several explanations, including post-transcriptional regulation, tubular remodeling, fibrosis, inflammatory remodeling, altered vascular or endothelial transcriptional state, or differences between plasma and tissue measurements. The present data do not distinguish among these possibilities.

Cell composition is the main interpretive limitation of this study. Because this analysis used bulk cortex RNA sequencing, apparent expression changes may reflect both within-cell transcriptional shifts and changing proportions of tubular, inflammatory, endothelial, and fibrotic populations. Loss of transcript-rich tubular epithelium, expansion of inflammatory infiltrates, fibrosis, endothelial rarefaction, or altered endothelial transcriptional state could influence genes such as VEGFA, HMOX1, FTL, NCOA4, and ALOX5. This limitation constrains the interpretation to bulk-tissue transcript-level associations.

The exploratory marker-set summaries provide context for this limitation but do not replace formal deconvolution. Mean z-scored log_2_(CPM+1) expression of conserved marker sets showed that macrophage/monocyte markers (CD68, CD163, and LYZ) were elevated in both CKD 1/2 and CKD 3/4 relative to controls, cytokine/NF-κB and fibroblast/myofibroblast marker sets increased with ordinal disease group, and proximal-tubule markers (SLC34A1, LRP2, and MIOX) decreased sharply in CKD 3/4 (Table S7). These patterns are consistent with inflammatory and fibrotic cortical remodeling, but they cannot quantify cell fractions or definitively assign ALOX5, VEGFA, FTL, or NCOA4 changes to specific cell populations. Cell-resolved or spatial transcriptomic studies will be needed to separate cellular abundance from within-cell transcriptional regulation.

The whole-transcriptome enrichment results are consistent with this calibrated interpretation. If a coherent ferroptosis program were the dominant cortical signal captured by bulk RNA sequencing, stronger agreement between the panel findings and ferroptosis-specific enrichment would be expected. Instead, immune and inflammatory processes dominated the advanced-stage transcriptome, while the 23-gene panel showed internally divergent stage-associated signals. This supports a model of overlapping inflammatory, redox, iron-handling, and hypoxia-response remodeling rather than a single defining mechanism.

This study has additional limitations. It was cross-sectional and therefore cannot establish temporality. Sample size was modest. With n = 21, the panel-level Spearman analyses were powered (80%, α = 0.05, two-sided) to detect only strong monotone associations (|ρ| ≳ 0.58); moderate associations may have been missed, confidence intervals for several stage associations were wide, and the relative ranking of candidate genes should be treated as provisional pending replication. A formal power analysis was not performed for the DESeq2 LRT or pairwise differential-expression analyses, whose power depends on dispersion, mean expression, filtering, model specification, and multiple-testing burden. The CKD groups were collapsed into CKD 1/2 and CKD 3/4 because the available sample size did not support stable inference across individual IRIS stages, so the results should not be interpreted as precise stage-specific trajectories. Descriptive composite estimates in CKD 3/4 were fragile to single-sample exclusion: leaving out one technically retained sample shifted the CKD 3/4 mean composite from 0.03 to 0.30 (Table S3), so late-stage descriptive estimates should be interpreted as provisional rather than as stable summary statistics. Individual-level demographic, treatment, comorbidity, serum creatinine, and symmetric dimethylarginine data were not available from the public deposit for the analytic subset, precluding covariate-adjusted analyses and representing systematic missing data that could introduce unmeasured confounding; the age comparison (*p* = 0.85) reported by Li et al. applies to the 23-cat source cohort, not the 21-cat analytic subset [5]. The statistical analysis plan was internally fixed before the review of the present expression results but was not externally time-stamped in a public registry. Finally, messenger RNA abundance does not establish protein stabilization, enzyme activity, cellular localization, iron phenotype, lipid-oxygenation injury, pathway activation, or cell death. Those questions will require orthogonal validation by tissue protein assays, iron mapping, lipid-oxygenation measurements, and cell-resolved transcriptomic or spatial approaches.

The strengths are the analysis-plan-guided panel, whole-transcriptome context, transparent reporting of significant and nonsignificant results, explicit handling of the unstable composite, and direct integration of exploratory marker-set and enrichment findings with the limitations of bulk RNA-seq. In practical terms, these data identify ALOX5-associated inflammatory/lipid-oxygenation signal and transcript-level HIF1A–VEGFA divergence as candidate patterns for prospective study in feline CKD. Although pharmacologic inhibition of 5-lipoxygenase reduces fibrosis and CKD progression in experimental models, the present bulk RNA-sequencing data do not justify therapeutic inference [15]. If validated, these transcript patterns may help define biologically distinct cortical response states rather than a single generic oxidative-injury mechanism.

## 5. Conclusions

In the renal cortex of cats with CKD, genes related to iron handling, lipid oxygenation, and hypoxia response showed bidirectional, gene-level change rather than coordinated activation of a single pathway. Increased ALOX5 and HIF1A together with decreased VEGFA, FTL, and NCOA4 were consistent with heterogeneous inflammatory/lipid-oxygenation, iron-handling, and hypoxia-response remodeling in bulk cortex. The absence of stage-associated GPX4 expression and ferroptosis-pathway enrichment means that the present transcriptomic data do not provide evidence sufficient to claim ferroptotic cell death, pathway activation, or cell-specific mechanism. Cell-composition shifts may contribute materially to these associations and should be resolved with cell-resolved approaches. These results identify ALOX5-associated inflammatory/lipid-oxygenation signal and transcript-level HIF1A–VEGFA divergence as candidate patterns for prospective validation in feline CKD.

## Figures and Tables

**Table 1:** Source-cohort demographics and analytic-sample distribution.

Characteristic	Overall Cohort	Control	CKD 1/2	CKD 3/4
Age, years (mean)	–	13.7	14.3	12.8
Sex, F/M	14/9	–	–	–
Breed, DSH/DLH	19/4	–	–	–
Cortex samples (primary)	–	6	8	7
Medulla samples (exploratory)	–	6	7	4
Excluded samples	–	0	1 cortex	1 medulla

Demographic data are from the full 23-cat source cohort reported by Li et al. and are not stratified to the retained analytic subset used here. Individual-level age, sex, breed, serum creatinine, and symmetric dimethylarginine values were not available for the retained subset from the public deposit. The excluded samples were SRR34712210 (CKD 1/2 cortex) and SRR34712180 (CKD 3/4 medulla). Dashes (–) indicate values not reported for that subgroup.

**Table 2:** Stage associations within the cortical panel (top genes).

Gene	Functional Group	Spearman ρ	Spearman q	LRT Adj. *p*
*VEGFA*	Hypoxia response	−0.81	<0.001	1.22 × 10^−7^
*FTL*	Iron handling	−0.78	<0.001	3.39 × 10^−4^
*ALOX5*	Lipid oxygenation/inflammation	0.77	<0.001	5.05 × 10^−3^
*NCOA4*	Ferritin handling	−0.72	0.001	6.12 × 10^−4^
*CISD1*	Iron handling	−0.63	0.010	0.054
*EGLN3*	Hypoxia response	−0.59	0.020	0.020
*HMOX1*	Redox/heme handling	0.56	0.028	0.077
*HIF1A*	Hypoxia response	0.54	0.035	7.10 × 10^−6^
*STEAP3*	Iron handling	0.53	0.037	1.23 × 10^−3^
*EGLN1*	Hypoxia response	−0.50	0.047	4.32 × 10^−3^

Top stage-associated genes from the 23-gene cortical panel; full 23-gene results are provided in Supplementary Table S1, and pairwise DESeq2 log_2_ fold changes with adjusted *p* values are provided in Supplementary Table S2a. Spearman ρ = correlation coefficient with ordinal disease group; Spearman q = Benjamini–Hochberg-adjusted *p* across all 23 per-gene panel-level correlations. LRT adj. *p* = panel-adjusted likelihood ratio test for any disease-group effect.

**Table 3:** Descriptive statistics for the equal-weight cortical composite.

Stage	n	Median Composite	IQR	Mean Composite	SD
Control	6	0.20	0.47	0.16	0.29
CKD 1/2	8	−0.09	0.51	−0.15	0.40
CKD 3/4	7	0.26	0.12	0.03	0.73

The equal-weight composite retains the earlier terminology used in the statistical analysis plan and is presented here as a descriptive secondary summary. Rounded values are shown to 2 decimal places in the manuscript text. Composite sensitivity summaries are provided in Supplementary Table S3, and within-arm heterogeneity in gene-level stage associations is shown in Figure S2.

**Table 4:** Pairwise effect sizes for the equal-weight cortical composite.

Comparison	Hedges’ g	95% CI
Control vs. CKD 1/2	0.80	−0.13, 2.13
Control vs. CKD 3/4	0.21	−1.65, 1.05
CKD 1/2 vs. CKD 3/4	−0.29	−2.44, 0.65

All bootstrap 95% confidence intervals cross zero, consistent with an unstable, low-information composite summary. These estimates are descriptive and not used for confirmatory inference.

## Data Availability

The raw RNA-seq data analyzed in this study are available from NCBI Gene Expression Omnibus under accession GSE303653. Manuscript-level supplementary outputs, DESeq2 contrast summaries, the statistical analysis plan, Supplementary Tables, figure source tables, and figure-generation code are available in Figshare at https://doi.org/10.6084/m9.figshare.32621199 (accessed on 29 April 2026). Derived count matrices not included in that archive are available from the author upon reasonable request.

## Supplementary Materials

The following supporting information can be downloaded at: https://www.mdpi.com/article/10.3390/vetsci13060604/s1, Supplementary Document S2. STROBE checklist for cross-sectional studies; Figure S1. Composite summary and high-influence-sample sensitivity; and Figure S2. Within-arm heterogeneity of gene-level stage associations. The statistical analysis plan, supplementary tables, figure source tables, and figure-generation code are deposited in the Figshare source package at https://doi.org/10.6084/m9.figshare.32621199 (accessed on 29 April 2026). Supplementary Document S1. Statistical Analysis Plan v1.2 (original analysis plan). Table S1. Full per-gene Spearman correlations for all 23 panel genes with ordinal disease group. Table S2. Whole-transcriptome differential-expression and likelihood ratio test summary, including enrichment query sizes and unmapped counts. Table S2a. Panel gene differential-expression results for all three pairwise contrasts. Table S2b. Panel gene likelihood ratio test results. Table S3. Sensitivity analysis for the equal-weight cortical composite with and without the technically retained high-influence sample. Table S4. Exploratory medulla descriptive statistics for the equal-weight composite. Table S5. g:Profiler enrichment results for CKD 3/4 versus control differentially expressed genes. Table S6. Sensitivity analyses including SLC7A11 inclusion, trimmed means, and weight perturbation. Table S7. Exploratory cell-composition marker analysis: mean z-scored log2(CPM+1) expression of conserved macrophage/monocyte, proximal-tubule, endothelial, fibroblast/myofibroblast, and cytokine/NF-kappaB marker sets by ordinal disease group in renal cortex.

## Appendix A. Detailed Materials and Methods

### Appendix A.1. Data Source and Analytical Cohort

This study used Cohort 2 from GSE303653, generated by Li et al. from cats with spontaneous CKD and healthy controls [5]. The public deposit contained 40 renal samples (22 cortex and 18 medulla samples) from 23 cats. Two samples were excluded before hypothesis testing because they met the analysis-plan-defined technical quality-control failure criteria stated in Materials and Methods (mapping rate < 35% OR duplication > 70% OR robust within-tissue principal-component-analysis outlier with Mahalanobis distance *p* < 0.01): one medulla sample (mapping rate, 21.6%; duplication, 80.6%) and one cortex sample (mapping rate, 32.2%; duplication, 73.5%) that also was a principal-component outlier. The retained dataset therefore comprised 38 samples from 22 cats: 21 cortex samples for the primary analysis and 17 medulla samples for exploratory summaries. Cortex group sizes were 6 controls, 8 CKD 1/2, and 7 CKD 3/4. Medulla group sizes were 6, 7, and 4, respectively. Stage assignments followed the source study classification, which was based on IRIS stages [3,5]. Because the public deposit did not include a complete individual-level covariate table for the analytic subset, demographic variables were reported from the source cohort description rather than re-estimated for the retained subset [5].

### Appendix A.2. RNA-Seq Processing and Panel Definition

Raw reads were quality-filtered with fastp and assessed with FastQC. Transcript quantification used Salmon against the *Felis catus* 9.0 reference genome (Ensembl release 113) [19]. The broader workflow followed nf-core/rnaseq conventions [18]. The 23-gene cortical panel was chosen before hypothesis testing and comprised 13 genes in an iron-handling/lipid-oxygenation arm (GPX4, ACSL4, FTH1, FTL, TFRC, HMOX1, NCOA4, SLC40A1, NFE2L2, CISD1, LPCAT3, STEAP3, and ALOX5) and 10 hypoxia-response genes (HIF1A, EPAS1, EGLN1, EGLN2, EGLN3, VEGFA, BNIP3, LDHA, SLC2A1, and HIF1AN). All 23 genes were detected in the cortex. The original statistical analysis plan referred to the equal-weight composite as the ferroptosis–HIF axis (FHA) score; that historical term is retained in Supplementary Document S1 because it reflects the analytical record, whereas the manuscript uses more specific biologic language when interpreting the results.

### Appendix A.3. Composite Computation and Cortex Analyses

For descriptive composite analyses, gene-level counts were converted to counts per million, transformed as log2(counts per million + 1), and standardized within tissue. The composite was calculated as 0.5 multiplied by the mean z-score of the 13-gene iron-handling/lipid-oxygenation arm plus 0.5 multiplied by the mean z-score of the 10-gene hypoxia-response arm. Arm scores were also summarized separately. Because the observed cortical composite pattern was nonmonotonic and sample size was limited, composite results were treated as descriptive rather than confirmatory and are reported as medians, interquartile ranges, means, standard deviations, and Hedges’ g with bootstrap 95% confidence intervals. All bootstrap confidence intervals used 10,000 nonparametric case-level resamples with random seed 20260301 and percentile confidence intervals. Gene-level stage associations were estimated with Spearman correlations and bootstrap confidence intervals. Whole-transcriptome differential expression used DESeq2 with the design formula ~ disease_stage in cortex [20]. Pairwise contrasts were CKD 1/2 versus control, CKD 3/4 versus control, and CKD 3/4 versus CKD 1/2, computed as default DESeq2 Wald tests using the unshrunken maximum-likelihood log2 fold change estimates (no lfcShrink was applied; |log2 fold change| > 1 was used as an effect-size threshold (low-count behavior is handled by DESeq2 independent filtering)). Stage-dependent expression across all three groups was evaluated with a likelihood ratio test against the reduced model ~ 1, with Benjamini–Hochberg adjustment within the LRT family. Significant genes for pairwise contrasts were defined as Benjamini–Hochberg-adjusted *p* < 0.05 within the contrast AND |log2 fold change| > 1. Functional enrichment of differentially expressed genes used g:Profiler with the g:SCS multiple-testing correction [21].

### Appendix A.4. Sensitivity Analyses and Exploratory Medulla Summary

The technically retained cortex sample with the most negative composite score was evaluated in a leave-one-out sensitivity analysis. Because that sample passed technical quality-control thresholds, it was retained in the primary analysis and reported separately only to show its effect on late-stage variance. Additional sensitivity analyses from the statistical analysis plan included SLC7A11 inclusion, trimmed means, and weight perturbation; those results are summarized in Supplementary Tables S3 and S6. Medulla analyses were limited to descriptive summaries because the CKD 3/4 medulla sample size was four and no inferential claims were planned for that tissue. All analyses were performed in R v4.4.3 using DESeq2 v1.44.0, Salmon v1.10.1, and g:Profiler (accessed on February 2026).
