# Supplementary material for: Iron-Handling, Lipid-Oxygenation, and Hypoxia-Response Gene Expression in the Renal Cortex of Cats with Chronic Kidney Disease: An Analysis-Plan-Guided Secondary Analysis
Source: Vet Sci. 2026 Jun 22;13(6):604. doi: 10.3390/vetsci13060604 (PMC13307564; doi:10.3390/vetsci13060604)
Supplement: Supplementary file 1 [file vetsci-13-00604-s001.zip › Supplementary Document S1.pdf]

Supplementary Document S1

Statistical Analysis Plan: Ferroptosis-HIF Axis (FHA) in Feline CKD

Version: 1.2 **Date:** 2026-03-01 **Amended:** 2026-03-17 **Supersedes:** SAP v1.1 **Dataset:** GSE303653 (Feline renal cortex and medulla RNA-seq) **Reference genome:** Felis_catus_9.0, Ensembl release 113 **Quantification:** Salmon pseudo-alignment via nf-core/rnaseq

Editorial note for submission package: This document is provided as the original prespecified analysis plan. The term 'ferroptosis-HIF axis (FHA)' is retained here as historical SAP terminology. In the manuscript, the findings are interpreted more narrowly as altered iron-handling, lipid-peroxidation-associated, and hypoxia-response gene expression because transcriptomic data alone do not establish ferroptotic cell death. Software and pipeline versions listed in Section 8 of this SAP reflect the planning-document state and may differ from the versions used in the completed run; the final manuscript Methods report the software versions actually used (nf-core/rnaseq v3.16.1, Salmon v1.10.1, fastp v0.23.4), as recorded in the nf-core software-versions metadata produced by that run, and supersede any version labels retained here.

1. Study Objective

To characterize transcriptional dysregulation of ferroptosis and HIF pathway genes in feline chronic kidney disease (CKD), using a composite gene expression score (FHA) applied to a publicly available RNA-seq dataset of feline renal tissue across IRIS CKD stages.

STROBE compliance: This secondary analysis of publicly deposited RNA-seq data follows the STROBE checklist for cross-sectional studies (Supplementary Document S2). Bias mitigation measures include pre-specified QC exclusion criteria (Section 2.2), prespecified no-go gates (Section 6), and a Track 1/Track 2 analytical firewall (Section 4.3). Potential confounders (age, sex, breed) are acknowledged but cannot be modeled as covariates given sample size constraints (n = 21 cortex); their distributions are reported descriptively in Table 1. The sample flow from

GEO deposit (23 cats, 40 samples) through QC exclusions to final analytic sets (21 cortex, 17 medulla) is documented in Section 2.1–2.3. Missing data: no imputation is performed; samples failing QC are excluded entirely (Section 2.2). Generalizability is limited to the source population (domestic cats with naturally occurring CKD evaluated at a single commercial research facility) and the specific transcriptomic platform (bulk RNA-seq of renal tissue).

1.1 Two-Track Design

- **Track 1 (FHA composite):** Descriptive mechanistic endpoint. Tier 3 – non-confirmatory. Reports effect sizes, bootstrap CIs, and nominal p-values with explicit power accounting.
- **Track 2 (whole-transcriptome):** Primary inferential discovery stream. DESeq2 under BH-FDR control, GO/KEGG enrichment, WGCNA co-expression.

1.2 Analysis Classification

Track 1: Tier 3 (descriptive). Monte Carlo power simulation (10,000 iterations, seed 20260301) demonstrated that no statistical test achieves 80% power at alpha = 0.05 with observed effect sizes (Cohen's f = 0.24, Cohen's d = -0.25 for Control vs CKD 3/4). Best achievable power: 12.6% (one-way ANOVA). The FHA composite is reported as a descriptive endpoint, not a confirmatory test.

Track 2: Inferential. DESeq2 stage contrasts with BH-FDR provide adequately powered discovery (2,686 DEGs identified in cortex CKD 3/4 vs Control at padj < 0.05 AND |log2FC| > 1).

1.3 Rationale for Proceeding Under Tier 3

This study proceeds despite low Track 1 power because: (a) it is a secondary analysis of a public dataset, not a prospective trial; (b) Track 2 provides adequately powered inferential discovery; (c) negative or inconclusive FHA results are scientifically informative and will be reported as a constrained finding; (d) the analysis framework and gating procedure have independent methodological value.

2. Study Design

2.1 Data Source

- **Accession:** GSE303653 (NCBI GEO)
- **Species:** Felis catus (domestic cat)
- **Tissue:** Renal cortex and medulla (paired from same animals where available)
- **Samples:** 40 total (from 23 cats in GEO deposit; 22 unique cats after QC, 21 with cortex), 38 usable after quality exclusions
- **Groups:** Control (healthy), CKD IRIS Stage 1/2, CKD IRIS Stage 3/4
- **Institutional provenance:** Nestlé Purina PetCare (IACUC protocols NT7427/NT7749)
- **Cohort demographics (Li et al. [13]):** 19 DSH / 4 DLH; 14 female / 9 male. Age by group: Control 13.7 y (mean), CKD 1/2 14.3 y, CKD 3/4 12.8 y (Kruskal-Wallis P = 0.85). Breed, sex, and age distributions are reported as aggregate statistics in Table 1.

2.2 Sample Exclusions (Pre-Analysis)

Two samples excluded during pipeline QC, prior to any hypothesis testing:

Sample	Reason
SRR34712180	21.6% mapping rate, 80.6% duplication (library failure)
SRR34712210	32.2% mapping rate, 73.5% duplication, PCA outlier (PC2 = 96.5)

2.3 Usable Sample Sizes

Tissue	Control	CKD 1/2	CKD 3/4	Total
Cortex	6	8	7	21 (from 21 unique cats)
Medulla	6	7	4	17 (from 17 unique cats)
**Total**	**12**	**15**	**11**	**38**

2.4 Tissue Scope

- **Cortex:** Primary analysis tissue (all tracks)
- **Medulla:** Exploratory only (n = 4 for CKD 3/4 insufficient for inference)

2.5 SRR34712199 Handling (Pre-Specified)

SRR34712199 (CKD 3/4, cortex, FHA = -1.607) is a statistical outlier. Handling is fully pre-specified:

Inclusion rule: Retained in primary analysis unless technical QC failure is met.

Technical QC failure thresholds (prespecified): mapping rate < 35% OR duplication > 70% OR robust PCA outlier (Mahalanobis distance p < 0.01 within tissue).

Influence diagnostics (applied post-hoc, exclusionary only if BOTH met): - Cook’s D > 4/n (i.e., > 0.57 for n = 7 within CKD 3/4 cortex) - Leave-one-out shift in group mean > 0.5 pooled SD

If influence thresholds are met but technical QC is not failed: 1. Sample is retained but triggers mandatory constrained interpretation 2. A with/without sensitivity table is provided in supplement 3. If LOO shift changes FHA group median by > 0.5 SD, both results reported as co-primary summaries 4. Discussion explicitly addresses whether this sample alters descriptive conclusions

This conservative retention policy is a deliberate design choice to preserve sample size at n = 7.

3. FHA Composite Score Definition

3.1 Formula

FHA = 0.5 * z_mean(ferroptosis_core) + 0.5 * z_mean(hif_core)

Where z_mean is the mean z-score of log2(CPM + 1) values across genes in each arm, with z-scores computed per gene across all samples within a tissue.

3.2 Gene Sets

Ferroptosis core (13 genes): GPX4, ACSL4, FTH1, FTL, TFRC, HMOX1, NCOA4, SLC40A1, NFE2L2, CISD1, LPCAT3, STEAP3, ALOX5

HIF core (10 genes): HIF1A, EPAS1, EGLN1, EGLN2, EGLN3, VEGFA, BNIP3, LDHA, SLC2A1, HIF1AN

All 23 genes confirmed detected at CPM ≥ 1 in 100% of samples (A1 detectability gate).

3.3 Excluded Genes

Gene	Reason	Gate
HAMP	0% detection (zero renal expression, hepatocyte-produced)	A1
SLC7A11	13.2% detection, not stage-dependent (Fisher p = 0.59), LOCO r = 0.996	Sprint 1a
VHL	Absent from Ensembl 113 annotation, not quantifiable without re-indexing	Sprint 1b
HJV	36.8% detection, hepatic co-receptor	A1
TFR2	15.8% detection, hepatic iron sensor	A1
EPO	7.9% detection, peritubular interstitial cells only	A1
IL6	10.5% detection	A1

3.4 Score Computation Procedure

1. Start from Salmon length-scaled counts (gene-level, tx2gene summarized)
2. Compute CPM: count_ij / (sum_j counts) * 1e6
3. Log-transform: log2(CPM + 1)
4. Z-score per gene across all samples within tissue

5. Ferroptosis arm = mean z-score of 13 ferroptosis genes per sample
6. HIF arm = mean z-score of 10 HIF genes per sample
7. FHA = 0.5 * ferroptosis arm + 0.5 * HIF arm

4. Analysis Plan

4.1 Track 1: FHA Composite (Descriptive Mechanistic Endpoint – Tier 3)

4.1.1 Preferred Descriptive Summaries

Due to variance heterogeneity across CKD stages (CKD 3/4 SD = 0.727, 2.5x Control SD):

- **Preferred:** Median and IQR by CKD stage and tissue
- **Secondary:** Mean and SD
- Boxplots of FHA score by IRIS stage
- Report individual arm scores (ferroptosis and HIF) separately
- Heatmap of all 23 FHA genes by sample, ordered by CKD stage (Figure 1A)
- Effect sizes: Hedge's g (bias-corrected) with bootstrap 95% CIs for all pairwise comparisons

4.1.2 Reference Statistical Tests (Non-Confirmatory)

All p-values in Track 1 are reported for reader context only, explicitly not used for confirmatory inference:

- Kruskal-Wallis test (assumption-appropriate omnibus for the observed non-monotonic pattern)
- Jonckheere-Terpstra trend test for ordinal CKD stage. **Note:** Observed FHA group means are non-monotonic (CKD 1/2 = -0.145 < CKD 3/4 = 0.029 < Control = 0.160). The JT

test assumes monotonic trend; results are reported for completeness but Kruskal-Wallis is the assumption-appropriate omnibus test for this pattern.

- Pairwise Welch t-tests (Control vs CKD 1/2, Control vs CKD 3/4, CKD 1/2 vs CKD 3/4)
- All tests accompanied by: “Power for this comparison is estimated at X% (Monte Carlo, 10,000 simulations); nominal p-values are not used for confirmatory inference.”

4.1.3 Correlation Analyses

- FHA score vs kidney injury markers (ACTA2): Spearman rho with 95% bootstrap CI (Figure 1C).
- GPX4 expression vs lipid peroxidation signature (ACSL4, LPCAT3, ALOX5 mean z-score): Spearman rho with 95% bootstrap CI (Figure 1D)
- HIF arm score vs CKD stage: boxplots with Jonckheere-Terpstra trend test (Figure 1B)

4.1.4 Per-Gene Analysis

Supplementary table: Spearman rho of each of the 23 FHA genes vs ordinal CKD stage, with bootstrap 95% CIs. This characterizes gene-level heterogeneity driving the composite pattern and identifies which genes contribute to the non-monotonic FHA pattern.

4.1.5 Power Accounting Statement

The following statement will accompany all Track 1 results:

The FHA composite score analysis is a descriptive mechanistic endpoint (Tier 3). Monte Carlo power simulation (N = 10,000, seed = 20260301, alpha = 0.05) using observed effect sizes (Cohen’s f = 0.24 for three-group ANOVA; Cohen’s d = -0.25 for Control vs CKD 3/4) estimated power at 12.6% for ANOVA and 6.7% for binary Welch t-test with the available sample sizes (Control n = 6, CKD 1/2 n = 8, CKD 3/4 n = 7 cortex). Nominal p-values are

reported for reader context and are not used for confirmatory inference. Absence of statistical significance does not constitute evidence against the hypothesis; power is insufficient to distinguish small effects from null effects.

4.1.6 Pre-Specified Non-Monotonicity Interpretation

The observed non-monotonic FHA pattern (CKD 1/2 more negative than CKD 3/4) is prespecified as an observation requiring interpretation. Three candidate biological explanations will be evaluated in the Discussion:

1. **Fibrotic burnout:** Advanced CKD fibrosis silences ferroptosis/HIF pathway expression, normalizing the composite toward control levels
2. **Compensatory response attenuation:** Early CKD activates compensatory ferroptosis defense (GPX4, NFE2L2 upregulation) that becomes heterogeneous in advanced disease
3. **Clinical stage misclassification:** IRIS staging may not perfectly align with molecular disease progression

A planned exploratory analysis (per-gene Spearman correlation with ordinal CKD stage, Section 4.1.4) will identify which genes drive the non-monotonic composite pattern.

4.2 Track 2: Whole-Transcriptome Analysis (Primary Inferential Discovery)

4.2.1 Differential Expression

- DESeq2 tissue-stratified analysis (already completed)
- Design: ~ disease_stage (within cortex, within medulla)
- Contrasts: CKD 1/2 vs Control, CKD 3/4 vs Control, CKD 3/4 vs CKD 1/2
- LRT omnibus test for any stage effect
- Significance: padj < 0.05 (Benjamini-Hochberg), |log2FC| > 1

- Pre-specified confounder sensitivity models: if batch or library-size effects suspected, re-run with covariates

4.2.2 Pathway Enrichment

- GO Biological Process and KEGG pathway enrichment on DEG lists
- Focus terms: ferroptosis (KEGG:map04216), HIF-1 signaling (KEGG:map04066), iron homeostasis, oxidative stress
- Method: g:Profiler (g:SCS multiple testing correction)
- Execution note: g:Profiler query sizes were smaller than DEG totals when some feline identifiers did not map to the enrichment namespace (CKD 1/2 vs Control 535 of 539; CKD 3/4 vs Control 2678 of 2686; CKD 3/4 vs CKD 1/2 249 of 250). These mapped query sizes are reported in Table S2.

4.2.3 Co-expression Analysis

- WGCNA on cortex samples (n = 21) – **DEFERRED.**
- Original specification retained for reference:
  - Signed hybrid network; soft-threshold selected by scale-free topology fit R^2 > 0.80
  - Minimum module size: 30 genes
  - Module preservation: 500 bootstrap resamples, Zsummary > 2 required
  - Test whether FHA genes cluster within CKD-correlated modules (convergent evidence)

4.3 Track 1 / Track 2 Firewall

Track 2 findings are reported as **convergent consistency** if they overlap Track 1 genes or pathways. Track 2 results never serve as independent confirmation of FHA composite score hypotheses.

Firewall rules: 1. Freeze and timestamp all Track 1 outputs before examining Track 2 pathway enrichment results 2. Document the analysis sequence with timestamps 3. Clearly label all Track 2 findings as "exploratory" in the manuscript 4. **Claim-decision matrix:** If Track 2 DESeq2 identifies N of 23 FHA component genes as differentially expressed under BH-FDR, this is reported as: "Track 2 identified N of 23 FHA component genes as differentially expressed, consistent with but not constituting independent confirmation of Track 1 FHA patterns, as both analyses derive from the same expression matrix." 5. Track 2 cannot be used to retrospectively "rescue" Track 1 FHA significance claims

5. Sensitivity Analyses

5.1 SLC7A11 Inclusion

Re-compute FHA with SLC7A11 added to ferroptosis arm (14 genes). Compare: - FHA vs FHA+SLC7A11 Pearson correlation (pre-computed: $r = 0.996$) - Repeat all Track 1 descriptive statistics and reference tests with FHA+SLC7A11

5.2 PCA-Derived Weights

Replace equal arm weights (0.5/0.5) with PC1 loadings from PCA of ferroptosis and HIF arm scores. Report whether PCA-derived FHA yields materially different group separation.

[Execution note: PCA-derived weighting remained exploratory and was not carried into the

current submission package because a finalized quantitative output was not versioned into the supplementary set.]

5.3 Outlier Sensitivity

SRR34712199 (CKD 3/4 cortex, FHA = -1.607): repeat all analyses with and without this sample per Section 2.5 governance. Report whether descriptive conclusions change. Note: removing this sample makes the non-monotonic pattern more pronounced (estimated CKD 3/4 mean shifts from 0.029 to +0.301).

5.4 Medulla Descriptive

Report FHA scores for medulla samples as supplementary material. No formal inference (n = 4 for CKD 3/4).

5.5 Trimmed Means

Report 10% trimmed means for CKD 3/4 group as an additional robust summary, given the higher CKD 3/4 SD. *[Execution note: with n = 7 cortex samples in CKD 3/4, a standard 10% trim removes zero observations from each tail, so the trimmed mean equals the arithmetic mean (0.029). This is reported in Table S6 for transparency rather than as a distinct robustness result.]*

6. No-Go Criteria (Quantitative)

6.1 Monotonicity Gate

Compute Kendall tau-b of FHA group medians vs ordinal CKD stage {1, 2, 3} with 10,000 bootstrap 95% CI.

Gate fails if: tau-b 95% CI upper bound < 0.1 (no detectable monotonic trend in FHA across disease stages).

6.2 Effect Size Gate

Compute Hedge's g (bias-corrected) for each pairwise comparison with bootstrap 95% CI.

Gate passes if: At least one pairwise |g| lower CI bound > 0.5 (medium effect detected).

6.3 Calibration (Pre-Registration Appendix A)

The operating characteristics of gates 6.1 and 6.2 were evaluated conceptually during planning via simulation with the following target scenarios: - True effect sizes: g = {0, 0.2, 0.5, 0.8, 1.0, 1.5} with n = 7 per group - 10,000 iterations per scenario - Target: ≥ 50% sensitivity for a true effect of g = 0.8 - Permutation-based CIs reported as sensitivity alternative to bootstrap CIs to mitigate undercoverage at n = 7

Fallback: If the 50% sensitivity target is unachievable at n = 7, report the maximum achievable sensitivity transparently and adjust the gate threshold to the most stringent level achieving ≥ 40% sensitivity.

6.4 Combined No-Go Decision

If the monotonicity gate fails (6.1) AND the effect size gate is not met (6.2):

Action: Terminate primary FHA hypothesis. Report as constrained negative finding with power accounting. Track 2 continues independently.

If FHA no-go triggers but Track 2 remains positive:

Conclusion: "FHA composite is constrained in this dataset; broader CKD transcriptomic dysregulation present."

6.5 Correlation Coherence

If Panel C (FHA vs injury markers) shows Spearman rho < 0.2 AND Panel D (GPX4 vs lipid peroxidation) shows rho > -0.2:

Action: Downgrade to “FHA composite lacks biological coherence in this dataset.” Report with full accounting.

7. Multiple Testing

- Track 1: No formal multiple testing correction (descriptive tier). All p-values reported as nominal, non-confirmatory.
- Track 2 DESeq2: Benjamini-Hochberg FDR (padj < 0.05)
- Track 2 enrichment: g:Profiler g:SCS correction
- Sensitivity analyses: reported as sensitivity, not independent tests

8. Software and Reproducibility

Tool	Version	Purpose
nf-core/rnaseq	3.x	RNA-seq pipeline
Salmon	(via nf-core)	Pseudo-alignment and quantification
DESeq2	(R 4.4)	Differential expression
g:Profiler	web API (2024-12)	Pathway enrichment (GO BP, KEGG)
WGCNA	(R 4.4)	Co-expression network analysis – **DEFERRED to Phase 2**
R	4.4	All statistical analyses
data.table	(R)	Data manipulation

All scripts archived in project repository. Random seed: 20260301. Monte Carlo/bootstrap iterations: 10,000. Software: nf-core/rnaseq v3.21.0, Nextflow v25.04.7, fastp v0.24.0, FastQC v0.12.1, Salmon v1.10.3, tximeta v1.20.1, DESeq2 v1.44.0, R v4.4.3, g:Profiler (web, accessed February 2026).

9. Reporting Standards

- STROBE-compliant observational study reporting; STROBE checklist provided as Supplementary Document S2
- All code and intermediate outputs versioned
- Negative and inconclusive results reported with equal rigor as positive findings
- Power limitations stated in Abstract, Methods, and Discussion
- Track 1 vs Track 2 distinction maintained in all sections

Post Hoc Addendum (added during manuscript finalization)

Two analyses not specified in the original SAP were performed during manuscript preparation and are reported as post hoc sensitivity analyses: (1) Benjamini-Hochberg correction of the 23 per-gene Spearman correlation P values, which retained 10 of 11 nominally significant genes at $q < .05$ (Table S1); and (2) PCA-derived composite weighting (SAP Section 5.2), which recovered equal arm loadings (PC1 = 88.4% of variance) and confirmed that weight perturbation across ferroptosis weights of 0.30 to 0.70 did not alter the non-monotonic composite pattern or yield a significant composite-stage association. Both analyses strengthen the manuscript's conclusions but were not prespecified and should be interpreted accordingly.
