# Supplementary material for: Iron-Handling, Lipid-Oxygenation, and Hypoxia-Response Gene Expression in the Renal Cortex of Cats with Chronic Kidney Disease: An Analysis-Plan-Guided Secondary Analysis
Source: Vet Sci. 2026 Jun 22;13(6):604. doi: 10.3390/vetsci13060604 (PMC13307564; doi:10.3390/vetsci13060604)
Supplement: Supplementary file 1 [file vetsci-13-00604-s001.zip › Supplementary Document S2 (STROBE checklist).pdf]

Supplementary Document S2

STROBE Statement — Checklist of Items for Cross-Sectional Studies

Manuscript: Iron-handling, lipid-oxygenation, and hypoxia-response gene expression in renal cortex of cats with chronic kidney disease: an analysis-plan-guided secondary analysis

Item No.	Recommendation	Reported in
**Title and Abstract**		
1(a)	Indicate the study's design with a commonly used term in the title or abstract	Title ('analysis-plan-guided secondary analysis'); Abstract ('analysis-plan-guided cross-sectional secondary analysis')
1(b)	Provide in the abstract an informative and balanced summary of what was done and what was found	Abstract (single-paragraph narrative summary, MDPI style; covers background, analysis-plan-guided study design, primary methods, principal findings, and interpretation)
**Introduction**		
2	Explain the scientific background and rationale for the investigation being reported	Introduction, paragraphs 1-2
3	State specific objectives, including any prespecified hypotheses	Introduction, paragraph 3
**Methods**		
4	Present key elements of study design early in the paper	Materials and methods, paragraph 1; Appendix 1: Data source and analytical cohort
5	Describe the setting, locations, and relevant dates, including periods of recruitment, exposure, follow-up, and data collection	Materials and methods, paragraph 1 (public RNA-seq dataset GSE303653; source study described in Li et al. 2025); Appendix 1
6(a)	Give the eligibility criteria, and the sources and methods of selection of participants	Materials and methods, paragraphs 1-2; Results: Sample characteristics and quality control
7	Clearly define all outcomes, exposures, predictors, potential confounders, and effect modifiers	Materials and methods, paragraphs 2-3; Appendix 1: Composite computation and cortex analyses
8	For each variable of interest, give sources of data and details of methods of assessment	Materials and methods, paragraphs 2-3; Appendix 1: RNA-seq processing and analysis-plan-defined panel
9	Describe any efforts to address potential sources of bias	Materials and methods, paragraph 1 (internal statistical analysis plan); Discussion, paragraphs 6-8
10	Explain how the study size was arrived at	Results: Sample characteristics and quality control; Discussion, paragraph 8

Item No.	Recommendation	Reported in
11	Explain how quantitative variables were handled in the analyses	Materials and methods, paragraphs 2-3; Appendix 1: Composite computation and cortex analyses
12(a)	Describe all statistical methods, including those used to control for confounding	Materials and methods, paragraph 3; Appendix 1
12(b)	Describe any methods used to examine subgroups and interactions	Materials and methods, paragraph 1 (exploratory medulla summary) and paragraph 3 (pairwise contrasts plus likelihood ratio test)
12(c)	Explain how missing data were addressed	Results: Sample characteristics and quality control (public subset limited to available deposited data; no imputation performed)
12(d)	If applicable, describe analytical methods taking account of sampling strategy	Not applicable (complete publicly deposited cohort subset after quality-control exclusions)
12(e)	Describe any sensitivity analyses	Results: Analysis-plan-defined composite summary; Appendix 1: Sensitivity analyses and exploratory medulla summary; Tables S3 and S6
**Results**		
13(a)	Report numbers of individuals at each stage of study — e.g., numbers potentially eligible, examined for eligibility, confirmed eligible, included in the study, and analysed	Results: Sample characteristics and quality control
13(b)	Give reasons for non-participation at each stage	Results: Sample characteristics and quality control (technical quality-control exclusions)
13(c)	Consider use of a flow diagram	Results: Sample characteristics and quality control (narrative sample flow)
14(a)	Give characteristics of study participants and information on exposures and potential confounders	Table 1; Discussion, paragraph 8
14(b)	Indicate number of participants with missing data for each variable of interest	Results: Sample characteristics and quality control; Table 1 note
15	Report numbers of outcome events or summary measures	Results: Tables 2-4; Supplementary Tables S1-S7
16(a)	Give unadjusted estimates and, if applicable, confounder-adjusted estimates and their precision	Results: Table 2 (correlation coefficients and 95% confidence intervals), Table 4 (Hedge's g and 95% confidence intervals), Supplementary Tables S1-S2b
16(b)	Report category boundaries when continuous variables were categorized	Materials and methods, paragraph 2 (control, CKD 1/2, CKD 3/4 stage groups from source study)
16(c)	If relevant, consider translating estimates of relative risk into absolute risk for a meaningful time period	Not applicable (cross-sectional transcriptomic study; no risk estimation)
**Discussion**		

Item No.	Recommendation	Reported in
17	Report other analyses done, such as analyses of subgroups and interactions, and sensitivity analyses	Results: Analysis-plan-defined composite summary; Appendix 1; Supplementary Tables S3-S6
18	Summarise key results with reference to study objectives	Discussion, paragraph 1
19	Discuss limitations of the study, taking into account sources of potential bias or imprecision	Discussion, paragraphs 6-8
20	Give a cautious overall interpretation of results considering objectives, limitations, multiplicity of analyses, results from similar studies, and other relevant evidence	Discussion, paragraphs 2-9
21	Discuss the generalisability (external validity) of the study results	Discussion, paragraphs 8-9
**Other information**		
22	Give the source of funding and the role of the funders for the present study and, if applicable, for the original study on which the present article is based	Title page: Funding and acknowledgments
