# Supplementary figures and images for: Iron-Handling, Lipid-Oxygenation, and Hypoxia-Response Gene Expression in the Renal Cortex of Cats with Chronic Kidney Disease: An Analysis-Plan-Guided Secondary Analysis
Source: Vet Sci. 2026 Jun 22;13(6):604. doi: 10.3390/vetsci13060604 (PMC13307564; doi:10.3390/vetsci13060604)

## Composite summary and sensitivity

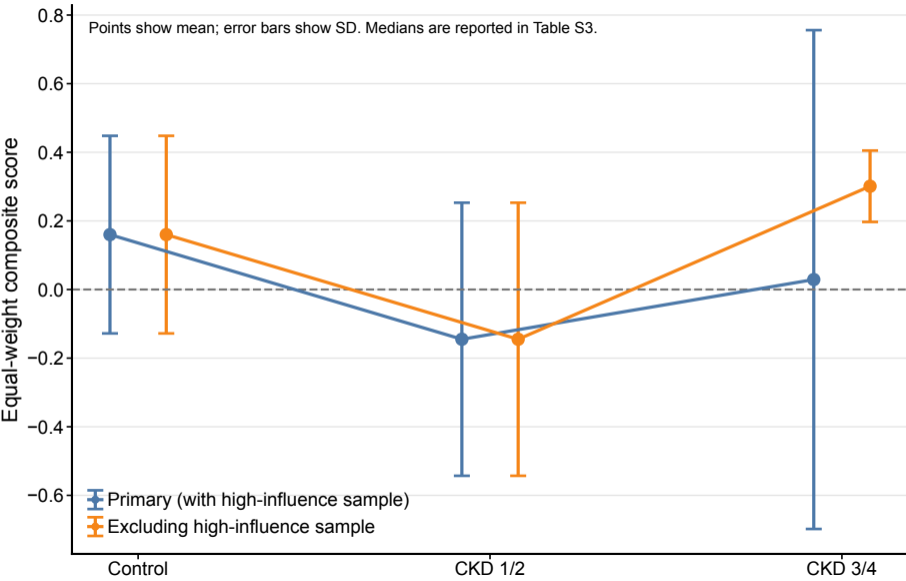

Supplement: Supplementary file 1 [file vetsci-13-00604-s001.zip › Figure S1.pdf]

# Within-arm heterogeneity of stage associations

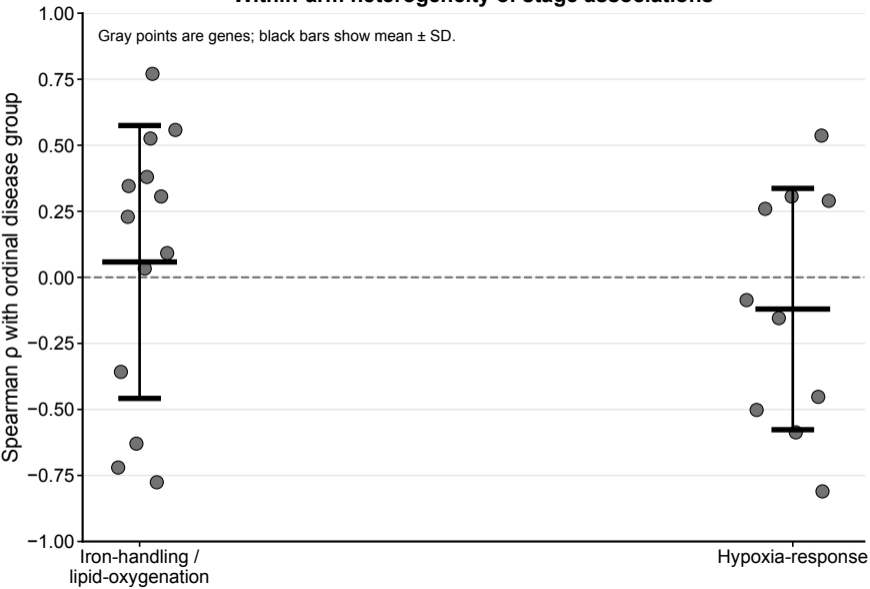

Supplement: Supplementary file 1 [file vetsci-13-00604-s001.zip › Figure S2.pdf]
